# Sexual functioning among breast cancer survivors and non‐cancer controls over 5 years post diagnosis: Pink SWAN

**DOI:** 10.1002/cam4.5433

**Published:** 2022-11-28

**Authors:** Nancy E. Avis, Sybil L. Crawford, Ellen B. Gold, Gail A. Greendale

**Affiliations:** ^1^ Department of Social Sciences & Health Policy Wake Forest University School of Medicine Winston‐Salem North Carolina USA; ^2^ Tan Chingfen Graduate School of Nursing UMass Chan Medical School Worcester Massachusetts USA; ^3^ Department of Public Health Sciences School of Medicine, University of California Davis California USA; ^4^ David Geffen School of Medicine University of California Los Angeles California USA

**Keywords:** breast cancer, menopause, sexual functioning, survivorship

## Abstract

**Purpose:**

To compare sexual functioning from diagnosis to 5 years post diagnosis among breast cancer survivors (BCS) and women without cancer (controls).

**Patients and Methods:**

Analyses included 118 BCS and 1765 controls from 20 years of the longitudinal Study of Women's Health Across the Nation (SWAN), a multiracial/ethnic cohort of mid‐life women assessed approximately annually from 1995 to 2015. Pink SWAN participants reported no cancer at SWAN enrollment and developed (BCS) or did not develop (controls) incident breast cancer after enrollment. Outcomes included: being sexually active or not, intercourse frequency, sexual desire, vaginal dryness, and pain with intercourse. Using longitudinal logistic regression, we compared BCS and controls on prevalence of sexual functioning outcomes with respect to years since diagnosis. In addition, we examined whether menopause transition stage, depressive symptoms, relationship satisfaction, vaginal dryness, or pain with intercourse modified the relation between breast cancer and sexual functioning outcomes.

**Results:**

Adjusting for partner status, both BCS and controls reported similar declines over time in being sexually active, sexual intercourse frequency, and sexual desire. Among sexually active women, more BCS than controls consistently reported vaginal dryness with significant differences between 2 and 4 years post‐diagnosis, and pain with intercourse, with statistically significant differences between 0.5 years post‐diagnosis to 2 years post‐diagnosis. Being post‐menopausal and reporting depressive symptoms were significant effect modifiers for pain with intercourse with both variables having positive and stronger associations with pain among the controls than among BCS.

**Conclusion:**

Except for more reporting of vaginal dryness and pain with intercourse among BCS, negative changes in sexual function during mid‐life were similar in those with and without breast cancer.

## Graphical abstract

This article compared sexual functioning over time from diagnosis to 5 years post‐diagnosis among breast cancer survivors (BCS) and women without cancer. Both BCS and controls reported similar declines over time in being sexually active. Among sexually active women, BCS and controls reported similar declines in frequency of sexual intercourse, and sexual desire. Except for more reporting of vaginal dryness and pain with intercourse among BCS, negative changes in sexual function during mid‐life were similar in women with and without breast cancer.

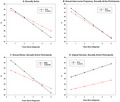

## 1 INTRODUCTION

Breast cancer is the most frequently occurring cancer among US women: about 3.8 million breast cancer survivors (BCS) are currently alive in the US (https://www.bcrf.org/breast‐cancer‐statistics‐and‐resources). After diagnosis and treatment, BCS frequently report sexual problems, which are important quality of life issues.^1^, ^2^, ^3^, ^4^, ^5^, ^6^, ^7^, ^8^, ^9^ The most frequently reported sexual problems include diminished sexual interest^10^, ^11^, ^12^ or desire,^1^, ^2^, ^4^, ^13^, ^14^ decreased arousal and lubrication,^1^, ^2^, ^4^, ^11^ and pain with vaginal penetration or intercourse.^2^, ^13^, ^14^

The vast majority of research on sexuality in BCS is cross‐sectional,^6^, ^9^, ^15^, ^16^, ^17^, ^18^, ^19^ based on women aged <50 years,^10^, ^11^, ^20^, ^21^, ^22^, ^23^ those diagnosed within the past year,^1^, ^7^, ^11^, ^17^, ^20^ and/or only sexually active BCS.^11^, ^17^, ^23^ We could identify only three studies that have measured sexual functioning over multiple time points post‐diagnosis, all of which have limitations. One study only examined being sexually active/inactive over 2 years.^12^ Another examined several domains of sexual problems (interest, enjoyment, orgasm, arousal, and pain) over 24 months, but this was among a select sample of premenopausal women with breast cancer who were receiving oral adjuvant endocrine treatment in a clinical trial.^24^ A third study looked at trajectories of change in sexual functioning over 5 years beginning shortly after diagnosis, but included only women aged 40 or younger who were sexually active.^23^ Given the limitations of these studies, the understanding of sexual functioning over time following a breast cancer diagnosis time still requires elucidation.

Because sexual functioning declines with age,^25^, ^26^ a longitudinal comparison group of women without cancer is needed to separate cancer effects from aging. Few studies of BCS have included such a comparison group, though some have used historical data from non‐cancer controls^11^, ^27^ or controls from another source, who may not be comparable.^6^, ^17^, ^22^ The few studies that have included control groups were cross‐sectional, had inconsistent results, or were of mixed cancer sites.^28^ We found no longitudinal studies of sexual functioning from breast cancer diagnosis to several years post‐diagnosis in comparison to that of healthy controls.

Previous research has found that older age,^12^, ^18^ past chemotherapy use,^4^, ^6^, ^7^, ^10^, ^12^, ^20^, ^29^ vaginal dryness or pain,^6^, ^8^, ^11^, ^12^, ^20^, ^24^, ^29^, ^30^ being post‐menopausal,^13^ depression,^8^, ^12^, ^18^, ^20^, ^21^, ^31^ and lower partner satisfaction^18^ were associated with greater sexual problems among BCS. However, except for chemotherapy, these variables also affect sexual function among women without cancer.^25^, ^26^, ^32^, ^33^ We identified only two studies that examined whether variables related to sexual functioning differed between BCS and controls, and both focused only on menopausal transition (MT) stage and had inconsistent results.^13^, ^34^

The Study of Women's Health Across the Nation (SWAN) Breast Cancer Survivors study (Pink SWAN) addresses many of the limitations of existing research. Pink SWAN, a SWAN substudy, focused on women who developed incident breast cancer, aged 45–69 years at diagnosis, during 22 years of SWAN follow‐up. Pink SWAN measured sexual functioning multiple times prior to and 5 years after breast cancer diagnosis, uniquely providing data on sexual functioning over time post‐diagnosis and addresses limitations of other studies that had multiple measures of sexual functioning by having 5 years of follow‐up, a wider age range, and more generalizable sample than did a previous study that also had multiple measures.^23^ SWAN also affords a comparator of women free from breast cancer and includes potential predictors of sexual functioning. This study aimed to: (1) compare sexual functioning between BCS and similarly aged women without breast cancer (controls) during the interval from breast cancer diagnosis to 5 years post diagnosis, and (2) determine whether associations of sexual functioning with variables previously identified as correlates of sexual function (MT stage, depression, vaginal dryness, pain, and relationship satisfaction), differed in BCS and controls.

## 2 METHODS

### 2.1 Sample and procedures

SWAN is a multi‐racial/ethnic cohort study characterizing biological and psychosocial changes occurring during the MT.^35^ From 1995 to 1997, seven clinical sites recruited study participants. All sites recruited non‐Hispanic white women for approximately half of their sample. In addition, each site recruited women from one of four racial/ethnic minorities (Black, Japanese, Hispanic or Chinese). All sites used a common protocol that was approved by each site's Institutional Review Board. All participants provided written informed consent.

Eligibility criteria included: age 42–52 years; an intact uterus and at least one ovary; not pregnant, lactating, using oral contraceptives or hormone therapy; and a menstrual cycle in the 3 months before screening. Participants were assessed in‐person at baseline and approximately annually through follow‐up visit 15 in 2015–6. Measures included: medical, reproductive and menstrual history; lifestyle and psychosocial characteristics; physical and psychological symptoms; and anthropometrics. Instruments were translated into Spanish, Japanese, and Cantonese.

Pink SWAN *BCS* are women who developed *incident breast cancer* following SWAN enrollment and had no other prior cancer. Pink SWAN *controls* are women who never developed breast cancer (before or after SWAN enrollment) and had no prior cancer at baseline. Beginning at visit 12, we requested medical records from women reporting incident breast cancer to verify diagnosis and treatment.

We identified 151 BCS and 2161 controls. Medical records were obtained for 110 BCS; breast cancers were confirmed in 104 (94.5% agreement). All self‐reports of a breast cancer diagnosis were adjudicated by a medical oncologist. As we have previously described,^36^ date of breast cancer diagnosis was the study's time “anchor” (i.e., time of diagnosis = 0). To assign a “pseudo date of diagnosis” in controls, we randomly assigned a visit as the first post‐diagnosis visit such that the distribution of first post‐diagnosis visits was comparable in BCS and controls, then set a random date between the last pre‐diagnosis visit and the first post‐diagnosis visit as the “pseudo date of diagnosis,” mirroring the process for BCS. A questionnaire assessing treatment‐related factors was mailed to BCS who were active in SWAN at follow‐up visit 15 (*N* = 130); 109 responded (83.8%).

### 2.2 Outcomes

SWAN measured sexual outcome variables at each study visit from baseline to visit 6 and at alternating study visits thereafter (follow‐up visits 8, 10, 12, 13, and 15). Women with and without partners completed a 20‐item, self‐administered questionnaire inquiring about sexual activity and function during the prior 6 months, which was returned to staff in a sealed envelope. The questionnaire was derived from several sources,^26^, ^37^, ^38^, ^39^ designed to cover important domains of sexual functioning, as previously described.^25^, ^32^, ^40^ Validation of the SWAN questions on five domains that matched both SWAN and the Female Sexual Functioning Inventory (FSFI)^41^ reported a high correlation (rho = 0.84).^42^ At SWAN visit 12, we administered the Short Personal Experiences Questionnaire (SPEQ)^43^ in addition to the SWAN questionnaire and report a high correlation between the SPEQ total score and the SWAN sexual functioning measure was 0.70, providing further concurrent validity of the SWAN measure.^31^ Domains assessed included:

*Sexually active/inactive* (*asked of all*). Women were considered sexually active if they responded “yes” to the following question: “During the past 6 months, have you engaged in sexual activities with a partner?” Women responding “no” were asked the reason(s) for inactivity: no partner, partner's physical problems, own physical problems.

Consistent with our previous work^25^, ^32^, ^40^ (Avis et al, 2017) and for ease of interpretation, we dichotomized responses for the following domains, to have approximately half the sample in each group:

*Desire* (*asked of all*) asked frequency of desire to engage in any form of sexual activity, either alone or with a partner (<weekly vs. ≥weekly).

*Sexual Intercourse* (*asked of sexually active*) inquired about frequency of sexual intercourse (< once/week vs. ~1 time per week or more), and *Pain with sexual intercourse (asked of sexually active*) assessed frequency of vaginal or pelvic pain during intercourse (at least sometimes vs. almost never or never). *Frequency of arousal* (asked of sexually active women) assessed frequency of feeling aroused during sexual activity (always/almost always vs. sometimes or less).

*Vaginal dryness* was asked of all women as part of a symptom list in the main SWAN interview form and assessed frequency of vaginal dryness in the past 2 weeks (categorized as any vs. no days).

To control for pre‐diagnosis sexual functioning, we created a pre‐diagnosis summary of each outcome, calculated as the percentage of visits at which the outcome was present during the 5 years prior to diagnosis.

### 2.3 Characteristics potentially related to sexual function

Candidate variables were selected based on previous associations with sexual functioning in SWAN^44^ and other studies of BCS.^4^, ^6^, ^8^, ^12^, ^13^, ^18^, ^31^ All variables were time‐varying. MT stages were pre/early peri‐menopause, late peri‐menopause, natural post‐menopause, surgical menopause (bilateral oophorectomy, with or without hysterectomy); undefinable due to pre‐menopausal hysterectomy (combined with the surgical category due to similar outcome distributions and small cell counts), and undefinable due to exogenous hormone use. Changes in MT stage from pre‐ to post‐diagnosis included: remained pre‐ or peri‐menopausal, transitioned to post‐menopause, and post‐menopausal pre‐diagnosis. Frequency of lubricant use, such as creams or jellies, to make sex more comfortable was categorized (at least sometimes vs. almost never or never).

Depressive symptoms, assessed by the Center for Epidemiologic Studies Depression (CESD) scale, was dichotomized to <16 versus ≥ 16.^39^ Degree of emotional relationship satisfaction with partner (very/extremely vs. moderately, slightly, or not at all) assessed satisfaction with partner relationship.

Additional covariates included: age at diagnosis, time‐varying marital status (married or living as married), and race/ethnicity.

Cancer‐related variables, ascertained from SWAN, medical records, and BCS questionnaire data were stage (0, I, II–III), ever chemotherapy, ever radiation, and medical treatments (selective estrogen receptor modulators [SERMs], other endocrine medications).

### 2.4 Statistical analyses

Pre‐ and post‐diagnosis characteristics of BCS and controls were compared using chi‐squared and t‐tests. For *Aim 1* analyses, we estimated a logistic regression model for each post‐diagnosis sexual functioning outcome as a function of four predictors: BCS/control status, years since diagnosis, the interaction of BCS/control status and years since diagnosis, and concurrent marital status with generalized estimating equations to accommodate within‐participant dependence.^45^ We adjusted for currently married/living as married as a potential confounder because BCS and controls differed significantly (see Table 1),

**TABLE 1:** Characteristics of breast cancer survivors (BCS; *N* = 118) and non‐breast cancer controls (*N* = 1765), pre‐ and post‐diagnosis

Characteristic	Time‐invariant		
	BCS	Controls	*p*‐Value
Age at diagnosis, years: Mean (SD)	56.2 (6.3)	55.6 (6.2)	0.27
Race/ethnicity: % (*N*)			0.29
White	54.2 (64)	48.6 (858)	
Black	24.6 (29)	26.2 (462)	
Hispanic	0.9 (1)	4.9 (86)	
Chinese	8.5 (10)	9.6 (170)	
Japanese	11.9 (14)	10.7 (189)	
Change in menopause transition stage, last visit prior to diagnosis visit to first after diagnosis visit			0.61
Not yet postmenopausal before and after diagnosis	32.5 (38)	34.7 (612)	
Not yet postmenopausal before diagnosis, postmenopausal after diagnosis	7.7 (9)	5.6 (99)	
Postmenopausal before and after diagnosis	59.8 (70)	59.7 (1051)	
Cancer‐related characteristics, breast cancer survivors only: % (*N*)			
Cancer stage:			–
0	27.8 (25)		
I	51.1 (46)		
II–III	21.1 (19)		
Missing	28		
Surgery:			–
None	1.0 (1)		
Lumpectomy or mastectomy	99.0 (103)		
*Missing*	*14*		
Any chemotherapy	38.3 (41)		–
*Missing*	*11*		
Any radiation therapy	70.2 (73)		–
*Missing*	*14*		
Any tamoxifen	33.1 (39)		–
Any endocrine treatment	37.3 (44)		–

	Time‐varying, last observed value within 5 years before diagnosis^a^			Time‐varying, first observed value within 5 years after diagnosis^a^		
	BCS	Controls	*p*‐Value	BCS	Controls	*p*‐Value
Menopause transition (MT) stage: % (N)			0.94			**0.002**
Pre‐ /early peri‐menopausal	32.5 (38)	30.7 (540)		17.0 (20)	24.8 (437)	
Late peri‐menopause	3.4 (4)	4.6 (81)		12.7 (15)	4.8 (85)	
Natural post‐menopause	55.6 (65)	53.8 (948)		63.6 (75)	58.7 (1036)	
Surgical menopause^b^	2.6 (3)	4.0 (70)		2.5 (3)	4.5 (80)	
Unknown, due to premenopausal hysterectomy^c^	1.7 (2)	1.9 (33)		1.7 (2)	2.1 (37)	
Unknown, due to systemic medication	4.3 (5)	5.1 (90)		2.5 (3)	5.1 (90)	
*Missing*	*1*	*3*		*0*	*0*	
Systemic medications precluding MT staging: % (*N*)			0.65			**<0.0001**
None	85.5 (100)	85.7 (1510)		51.7 (61)	85.8 (1514)	
Non‐systemic	2.6 (3)	1.3 (22)		0.0 (0)	1.6 (29)	
Estrogens and/or progestins	12.0 (14)	12.5 (220)		5.9 (7)	12.1 (213)	
Selective estrogen receptor modulators	0.0 (0)	0.4 (7)		20.3 (24)	0.5 (8)	
Antineoplastic hormones	0.0 (0)	0.0 (0)		22.0 (26)	0.0 (0)	
Gonadotropin releasing hormones	0.0 (0)	0.2 (3)		0.0 (0)	0.1 (1)	
*Missing*	*1*	*3*		*0*	*0*	
Self‐reported health: % (*N*)			0.66			**0.005**
Excellent	18.0 (21)	15.2 (268)		3.4 (4)	13.7 (242)	
Very good	40.2 (47)	38.4 (676)		35.6 (44)	39.4 (695)	
Good	31.6 (37)	32.7 (576)		41.9 (49)	31.5 (555)	
Fair/Poor	10.3 (12)	13.7 (241)		17.1 (20)	15.4 (272)	
*Missing*	*1*	*3*		*1*	*1*	
Currently married /living as married: % (*N*)	49.6 (58)	63.2 (1114)	**0.004**	49.2 (58)	62.0 (1093)	**0.006**
*Missing*	*1*	*4*		*0*	*1*	
Lubricant use at least sometimes, women sexually active with partner only: % (*N*)	38.0 (27)	32.6 (389)	0.36	51.7 (31)	37.4 (393)	**0.03**
Very emotionally satisfied, women sexually active with partner only: % (*N*)	51.4 (38)	52.1 (634)	0.91	64.1 (41)	55.6 (604)	0.20

*Note*: All data presented as % (*n*) or mean (SD). *p* Values <0.05 are bolded.

Abbreviation: SD, standard deviation.

^a^ For controls, a pseudo date of diagnosis was randomly assigned to match the corresponding distribution in BCS.

^b^ Surgical menopause defined as the occurrence of bilateral oophorectomy with or without hysterectomy.

^c^ For subsequent analyses, the unknown due to premenopausal hysterectomy was combined with surgical menopause; see Methods for rationale.

After examining LOESS plots^40^ of each outcome from pre‐ to post‐diagnosis (not shown) and testing linear versus non‐linear model fits, the more complex models did not improve fit; we, therefore, used a linear term for years since diagnosis. Because questions regarding sexual functioning covered the past 6 months, we excluded measurements made within 6 months of diagnosis to avoid overlap with pre‐diagnosis functioning. We also omitted observations occurring after a breast cancer recurrence in BCS and after a new cancer in both groups.

Analyses of sexual activity, desire, and vaginal dryness included all measurements 0.5–5 years post‐diagnosis, and analyses of other outcomes included only observations in this interval at which concurrent partnered sexual activity was reported. Additional analyses for desire and vaginal dryness included only observations with concurrent partnered sexual activity. Participants with no observed sexual functioning outcomes in the interval 0.5–5 years post‐diagnosis (*N* = 429; 21.9% of BCS and 18.3% of controls, *p* = 0.28 for difference between BCS and controls) were excluded from all analyses; the proportion excluded did not differ between BCS and controls. Visits with missing data for an outcome and/or predictor were excluded from the analysis for the outcome being modeled.

Associations of outcomes with years since diagnosis were expressed as per‐year adjusted odds ratios (aOR). Model‐based estimated outcome prevalences were compared for BCS and controls at 0.5, 1, 2, 3, 4, and 5 years post‐diagnosis. Sensitivity analyses adjusted for age, pre‐diagnosis outcome summaries, pain and dryness outcomes, and lubricant use. In BCS only, we used the same methodology to estimate associations of cancer‐specific variables with outcomes.

In *Aim 2* analyses of effect modification, we tested whether associations between selected characteristics and sexual function differed between BCS and controls. *Aim 2* analyses added two additional predictors to Aim 1 models: an hypothesized effect modifier and its interaction with BCS/control status; a separate model was run for each hypothesized effect modifier and each outcome. Characteristics examined were MT stage (dichotomized as post‐menopausal/BSO/hysterectomy vs. all other), vaginal dryness, pain, depressive symptoms, and emotional satisfaction.

Analyses used SAS 9.4 (SAS Institute Inc.). *p*‐Values ≤0.05 were considered statistically significant.

## 3 RESULTS

### 3.1 Sample characteristics

Of the 151 BCS and 2161 controls meeting inclusion criteria, 118 BCS and 1765 controls had at least 1 visit in the interval 6 months through 5 years post‐diagnosis and were included in analyses. BCS and controls did not differ on age, race/ethnicity, or pre‐diagnosis self‐reported health (Table 1). Both pre‐ and post‐diagnosis, BCS were significantly less likely than controls to be partnered (*p* < 0.01). Post‐diagnosis, BCS reported worse health (*p* < 0.01), and controls were almost twice as likely as BCS to be pre‐/early peri‐menopausal with no exogenous hormone use. BCS did not differ from controls in use of lubricants pre‐diagnosis but were significantly more likely to report use post‐diagnosis (*p* = 0.03). Most BCS were diagnosed at cancer stage I. No participants had metastatic cancer. Almost all BCS (99.0%) had surgery; 38.3% had chemotherapy, and 70.2% had radiation. The mean age at diagnosis/pseudo‐diagnosis was 56.2 years for BCS and 55.6 years for controls, *p* = 0.27. Of the 118 BCS, only one woman (0.9%) had a completely missed study visit post‐diagnosis, and among the 1765 controls, no one completely missed a study visit post‐pseudo‐diagnosis. Among these participants, 7.68% were missing sexual activity, and among those reporting sexual activity, the percent of missing data ranges from 0.50% (for dryness) to 6.02% (for pain). Loss to follow‐up (due to death or study drop‐out) was 20.3% (*N* = 24) among BCS and 10.1% (*N* = 178) among controls.

### 3.2 Sexual functioning over time

Table 2 presents results from longitudinal GEE logistic regressions, focusing on the BCS‐control differences over time, adjusting for concurrent marital status. For each sexual functioning outcome, we compared BCS and control participants regarding within‐woman change over time in sexual functioning, expressed in terms of the per‐year odds ratio; an odds ratio above 1 indicates an increase over time in the prevalence of the outcome, whereas an odds ratio below 1 indicates a decrease over time. The p‐value in the fourth column of Table 2 indicates the statistical significance of the BCS‐control difference in the per‐year odds ratio, that is, whether prevalence of the outcome is changing over time at a significantly different pace for the two groups. In addition, we compared BCS and control participants regarding estimated outcome prevalence at 0.5, 1, 2, 3, 4, and 5 years post‐diagnosis.

**TABLE 2:** Per‐year adjusted odds ratio (95% CI) for years since diagnosis and model‐estimated prevalences by breast cancer survivor (BCS) or control status, adjusted for concurrently married/living as married

Sexual Functioning Outcome	Per‐year adjusted odds ratio (aOR) and 95% CI			Prevalence, % (standard error)			
	BCS	Controls	*p*‐Value^a^	Years since diagnosis	BCS	Controls	*p*‐Value^b^
Sexually active with partner in past 6 months, all visits^c^	0.91 (0.78, 1.05)	0.93 (0.89, 0.97)**	0.71	0.5	64.37 (5.61)	63.62 (1.56)	0.90
				1	63.22 (5.19)	62.80 (1.41)	0.94
				2	60.87 (4.66)	61.14 (1.23)	0.96
				3	58.47 (4.74)	59.45 (1.25)	0.84
				4	56.03 (5.46)	57.74 (1.49)	0.76
				5	53.56 (6.65)	56.02 (1.88)	0.72
Intercourse at least weekly in past 6 months, in visits at which participant reported being sexually active^d^	0.88 (0.69, 1.12)	0.89 (0.84, 0.95)***	0.90	0.5	53.92 (8.43)	52.35 (1.98)	0.86
				1	52.33 (7.51)	50.96 (1.74)	0.86
				2	49.12 (6.35)	48.17 (1.45)	0.88
				3	45.93 (6.54)	45.39 (1.49)	0.94
				4	42.77 (7.92)	42.64 (1.84)	0.99
				5	39.66 (9.89)	39.94 (2.34)	0.98
Desire at least weekly in past 6 months, all visits^e^	0.89 (0.75, 1.06)	0.93 (0.89, 0.98)**	0.61	0.5	36.37 (5.61)	38.03 (1.48)	0.78
				1	35.06 (4.92)	37.24 (1.30)	0.67
				2	32.49 (4.08)	35.68 (1.07)	0.46
				3	30.03 (4.14)	34.15 (1.08)	0.35
				4	27.68 (4.87)	32.65 (1.29)	0.35
				5	25.44 (5.88)	31.19 (1.61)	0.37
Any vaginal dryness in past 2 weeks, all visits^f^	1.05 (0.91, 1.21)	1.01 (0.97, 1.04)	0.58	0.5	34.86 (5.01)	31.26 (1.26)	0.48
				1	35.40 (4.52)	31.33 (1.14)	0.37
				2	36.51 (3.85)	31.46 (0.97)	0.19
				3	37.63 (3.84)	31.60 (0.97)	0.12
				4	38.76 (4.54)	31.73 (1.12)	0.12
				5	39.90 (5.73)	31.87 (1.39)	0.16
Desire at least weekly in past 6 months among visits at which participants reported being sexually active^g^	0.90 (0.71, 1.13)	0.94 (0.89, 0.999)*	0.69	0.5	56.81 (7.55)	53.13 (1.98)	0.64
				1	55.48 (6.67)	52.39 (1.74)	0.66
				2	52.79 (5.60)	50.90 (1.43)	0.74
				3	50.08 (5.93)	49.41 (1.47)	0.91
				4	47.38 (7.48)	47.91 (1.85)	0.94
				5	44.69 (9.64)	46.43 (2.40)	0.86
Any vaginal dryness in past 2 weeks among visits at which participants reported being sexually active^h^	1.12 (0.94, 1.33)	1.08 (1.02, 1.15)**		0.5	46.69 (7.00)	35.11 (1.88)	0.10
				1	48.07 (6.45)	36.04 (1.66)	0.06
				**2**	**50.82 (5.83)**	**37.92 (1.37)**	**0.03**
				**3**	**53.57 (6.00)**	**39.84 (1.42)**	**0.03**
				**4**	**56.30 (6.89)**	**41.79 (1.81)**	**0.04**
				5	58.99 (8.20)	43.76 (2.40)	0.08
Pain with intercourse at least sometimes, visits at which participants reported being sexually active^i^	0.92 (0.74, 1.13)	1.08 (1.02, 1.15)**	0.13	0.5	**49.04 (8.08)**	**27.71 (1.76)**	**0.006**
				1	**47.94 (7.26)**	**28.54 (1.58)**	**0.006**
				2	**45.74 (6.17)**	**30.22 (1.35)**	**0.01**
				3	43.55 (6.12)	31.97 (1.40)	0.054
				4	41.39 (7.09)	33.76 (1.76)	0.28
				5	39.26 (8.65)	35.61 (2.32)	0.68
Always/almost always aroused in past 6 months among visits at which participants reported being sexually active^j^	1.18 (0.97, 1.44)	0.95 (0.90, 1.00)	**0.04**	0.5	61.76 (7.08)	64.43 (1.82)	0.71
				1	63.68 (6.36)	63.87 (1.64)	0.98
				2	67.39 (5.50)	62.72 (1.41)	0.42
				3	70.90 (5.47)	61.56 (1.45)	0.12
				4	**74.17 (6.02)**	**60.38 (1.75)**	**0.05**
				5	**77.19 (6.78)**	**59.20 (2.23)**	**0.03**

*Note*: GEE binomial logistic regression for sexual functioning outcomes, with predictors BCS‐control status, years since diagnosis, and their interaction; also adjusted for concurrently married/living as married. *p* Values <0.05 are bolded.

Abbreviations: BCS, breast cancer survivors; GEE, generalized estimating equation.

* *p* < 0.05

** *p* < 0.01

*** *p* < 0.001.

^a^ *p*‐Value for BCS versus controls difference in per‐year OR.

^b^ *p*‐Value for BCS versus controls difference in estimated outcome prevalence.

^c^ 115 observations from 57 BCS; 1941 observations from 1018 controls.

^d^ 124 observations from 62 BCS; 2046 observations from 1064 controls.

^e^ 217 observations from 118 BCS; 3336 observations from 1764 controls.

^f^ 121 observations from 60 BCS; 1985 observations from 1043 controls.

^g^ 223 observations from 118 BCS; 3436 observations from 1761 controls.

^h^ 315 observations from 116 BCS; 5028 observations from 1761 controls.

^i^ 123 observations from 62 BCS; 2054 observations from 1068 controls.

^j^ 124 observations from 62 BCS; 2053 observations from 1069 controls.

#### 3.2.1 Any/frequency of sexual activity

BCS reported a steady, but non‐statistically significant, decline in being sexually active (Table 2) from 64.4% at 6 months following diagnosis to 53.6% at 5 years post‐diagnosis (aOR per year, 0.91; 95% CI, 0.78–1.05). Importantly, controls showed a similar decline, from 63.6% at 6 months after pseudo‐diagnosis to 56.0% 5 years later (aOR per year, 0.93; 95% CI, 0.89–0.97; *p* = 0.71 for BCS‐control difference). BCS and controls also had similar declines in frequency of intercourse with time since diagnosis/pseudo‐diagnosis.

#### 3.2.2 Desire and vaginal dryness, regardless of reporting of being sexually active

Including all visits (regardless of reporting being sexual activity), desire declined similarly among BCS and controls (per‐year aOR, 0.89; 95% CI, 0.75–1.06 and 0.93; 95% CI, 0.89–0.98, respectively). Vaginal dryness prevalence increased for both BCS and controls, with a slightly steeper but statistically non‐significant rise and higher prevalence in BCS than in controls (per‐year aOR, 1.05; 95% CI, 0.91–1.21 and 1.01; 95% CI, 0.97–1.04 respectively).

#### 3.2.3 Desire, vaginal dryness and pain with intercourse, at visits reporting sexual activity

Including only visits at which participants reported being sexually active, prevalence of sexual desire was higher than for all vists, but otherwise patterns were consistent with results for all visits: BCS and controls both reported declines in desire over time. Per‐year increase in vaginal dryness was also similar for BCS and controls, and prevalence was consistently higher in BCS throughout. However, in contrast to all visits, BCS‐control differences were greater and statistically significant between 2 and 4 years post‐diagnosis. BCS also had a consistently higher prevalence of pain with intercourse than controls, differing significantly through 2 years post‐diagnosis.

Results of sensitivity analyses adjusting for age, pre‐diagnosis outcome summaries, and lubricant use were consistent with those presented (data not shown).

### 3.3 Cancer‐related variables

Among BCS, at visits at which women reported being sexually active, desire was lower in stages II–III than stage 0 (aOR, 0.17; 95% CI, 0.03–0.87, *p* = 0.03), and receipt of radiation therapy was significantly related to greater frequency of intercourse (aOR, 4.99; 95% CI, 1.60–15.58, *p* = 0.0057) (data not shown).

### 3.4 Effect modification by BCS‐control status

Previously identified correlates (depressive symptoms, vaginal dryness, pain with intercourse, emotional satisfaction with partner relationship, and pre‐vs. post‐menopausal) were significantly associated with at least one sexual functioning outcome in analyses of main effects (Table S1). However, few interactions between these characteristics and BCS‐control status were statistically significant (*p* < 0.05) (Table 3). Statistically significant interactions with BCS‐control status occurred only in analyses of visits in which women reported being sexually active; being post‐menopausal and having elevated depressive symptoms were significantly positively associated with pain with intercourse only in controls (*p* = 0.02 and 0.03 respectively). Being very emotionally satisfied was significantly more strongly associated with arousal in controls than in BCS (*p* = 0.02).

**TABLE 3:** Effect modification by BCS‐control status, adjusted for concurrently married/living as married

Outcome/Characteristic	Adjusted Odds Ratio (95% CI)		*p*‐value for interaction with BCS‐control status
	BCS	Controls	
Sexually active with partner in past 6 months, all visits			
Post‐menopausal	0.90 (0.36, 2.25)	0.51 (0.42, 0.61)****	0.23
Any vaginal dryness in past 2 weeks	1.67 (0.97, 2.85)	1.44 (1.23, 1.69)****	0.61
Depressive symptoms	1.31 (0.51, 3.37)	0.87 (0.71, 1.06)	0.40
Intercourse at least weekly in past 6 months, among visits at which participants reported being sexually active			
Post‐menopausal	1.06 (0.47, 2.51)	0.66 (0.54, 0.81)****	0.30
Any vaginal dryness in past 2 weeks	1.24 (0.73, 2.10)	1.07 (0.89, 1.28)	0.60
Pain with intercourse at least sometimes	0.62 (0.34, 1.11)	0.58 (0.46, 0.73)****	0.85
Very emotionally satisfied	1.58 (0.73, 3.46)	2.22 (1.83, 2.69)****	0.41
Depressive symptoms	0.85 (0.46, 1.57)	0.84 (0.64, 1.10)	0.97
Desire at least weekly in past 6 months, all visits			
Post‐menopausal	0.85 (0.38, 1.88)	0.53 (0.45, 0.63)****	0.27
Any vaginal dryness in past 2 weeks	1.29 (0.77, 2.17)	1.03 (0.89, 1.20)	0.42
Depressive symptoms	1.14 (0.56, 2.32)	0.78 (0.64, 0.96)*	0.32
Any vaginal dryness in past 2 weeks, all visits			
Post‐menopausal	1.18 (0.59, 2.37)	1.44 (1.21, 1.71)****	0.59
Depressive symptoms	1.23 (0.69, 2.19)	1.15 (0.98, 1.36)	0.83
Desire at least weekly in past 6 months, among visits at which participants reported being sexually active			
Post‐menopausal	0.78 (0.31, 1.93)	0.69 (0.56, 0.85)***	0.81
Any vaginal dryness in past 2 weeks	0.77 (0.41, 1.45)	0.89 (0.74, 1.07)	0.67
Pain with intercourse at least sometimes	0.37 (0.16, 0.87)*	0.55 (0.44, 0.68)****	0.39
Very emotionally satisfied	1.73 (0.96, 3.12)	2.21 (1.82, 2.69)****	0.44
Depressive symptoms	0.92 (0.35, 2.44)	0.68 (0.52, 0.89)**	0.55
Any vaginal dryness in past 2 weeks among visits at which participants reported being sexually active			
Post‐menopausal	1.20 (0.52, 2.75)	1.92 (1.54, 2.40)****	0.28
Pain with intercourse at least sometimes	6.61 (2.63, 16.61)****	3.39 (2.74, 4.21)****	0.17
Very emotionally satisfied	1.12 (0.51, 2.46)	0.75 (0.62, 0.90)**	0.33
Depressive symptoms	1.33 (0.2, 3.39)	1.21 (0.93, 1.56)	0.84
Pain with intercourse at least sometimes among visits at which participants reported being sexually active			
Post‐menopausal	0.71 (0.32, 1.58)	1.98 (1.55, 2.53)****	**0.02**
Any vaginal dryness in past 2 weeks	4.92 (2.05, 11.83)***	3.09 (2.51, 3.80)****	0.31
Very emotionally satisfied	0.93 (0.44, 1.99)	0.64 (0.52, 0.79)****	0.35
Depressive symptoms	0.39 (0.12, 1.32)	1.51 (1.15, 1.98)**	**0.03**
Always / almost always aroused in past 6 months among visits at which participants reported being sexually active			
Post‐menopausal	0.93 (0.41, 2.08)	0.63 (0.51, 0.78)****	0.37
Any vaginal dryness in past 2 weeks	1.04 (0.42, 2.53)	0.66 (0.54, 0.79)****	0.32
Pain with intercourse at least sometimes	0.29 (0.09, 0.91)*	0.49 (0.39, 0.60)****	0.38
Very emotionally satisfied	1.97 (1.07, 3.63)*	4.23 (3.40, 5.27)****	**0.02**
Depressive symptoms	0.28 (0.10, 0.79)*	0.74 (0.57, 0.97)*	0.07

*Note*: Generalized estimating equation (GEE) binomial logistic regression for sexual functioning outcomes, with predictors BCS‐control status, years since diagnosis, BCS‐control status × years since diagnosis, characteristic of interest, and BCS‐control status × characteristic of interest; adjusted for concurrently married/living as married.

*p* values < 0.05 are bolded.

* *p* < 0.05

** *p* < 0.01

*** *p* < 0.001

**** *p* < 0.0001.

## 4 DISCUSSION

This study found that some changes in sexual functioning previously attributed to breast cancer were also present in midlife women without cancer. Both groups showed a decline over time since diagnosis/pseudo‐diagnosis in being sexually active, sexual intercourse frequency, and sexual desire, and an increase in vaginal dryness. Time since diagnosis was important for BCS‐control differences for vaginal dryness and pain in visits in which women reported being sexually active. Characteristics, such as vaginal dryness, related to sexual functioning among BCS, were similarly related to sexual functioning among controls. These BCS‐control similarities highlight the importance of a non‐cancer control group and the value of multiple longitudinal assessments for at least 5 years, in understanding sexual functioning following a breast cancer diagnosis.

The percentage of BCS who were sexually active over 5 years post‐diagnosis was similar to that of controls, as was the rate of decline. The per‐year adjusted odds ratios were similar in both groups, but significant only for the controls due to their larger sample size and more narrow confidence intervals. The one other study that examined being sexually active/inactive over multiple times^12^ found very slight, but not significant, increases in being sexually active from close to diagnosis to 18 months later. However, a high percentage of women with BCS in this study were still receiving treatment at the initial assessment. This suggests that time since diagnosis, treatment status, and age may all be related to whether BCS are sexually active post‐diagnosis. Among sexually active women, no difference was observed between BCS and controls in sexual intercourse frequency with both groups exhibiting a decline. Some studies have reported lower frequency of intercourse among BCS compared to controls,^13^, ^16^ but, like our results, analyses that controlled or matched for age did not find lower sexual activity rates in BCS.^9^, ^15^, ^18^, ^34^ Given that sexual activity declines with age,^25^, ^34^ our results underscore the importance of an age‐matched comparator of women without cancer, a finding also demonstrated in the English Longitudinal Study of Aging of mixed cancer sites.^28^

In addition to frequency of sexual intercourse, we also found a non‐significant decline in desire and an increase in vaginal dryness among BCS. The specific timing of these declines varies somewhat from that of Ribi and colleagues,^24^ but this may be due to differences in the timing of assessments in relation to diagnosis and treatment. Although some studies have found lower desire or interest among BCS,^8^, ^16^ we found that desire decreased in women with and without cancer, again demonstrating the need for the control group. Consistent with other findings, despite greater use of lubricants, BCS who were sexually active were more likely than controls to report experiencing vaginal dryness in the first few years following diagnosis,^8^, ^34^ and significantly more reported pain or discomfort with intercourse^9^, ^13^, ^16^, ^17^, ^18^ than controls in the first 2 years following diagnosis. However, the difference between groups diminished over time for both outcomes.

We found little evidence for effect modification by BCS‐control status of characteristics previously related to sexual functioning among BCS (MT stage, depressive symptoms, vaginal dryness, pain, relationship satisfaction)^6^, ^8^, ^11^, ^12^, ^13^, ^18^, ^21^, ^24^, ^29^, ^30^, ^31^ and most outcomes. The exceptions were that post‐menopausal status and depressive symptoms were both positively, and more strongly related to pain with intercourse among controls.

In contrast to others,^6^, ^12^ we did not find a relationship between chemotherapy and sexual function among BCS, possibly because the majority of our sample either remained premenopausal or were already postmenopausal when diagnosed, or because we did not have timing of chemotherapy. Other studies have shown chemotherapy is most relevant when it induces menopause among previously premenopausal women.^6^, ^11^, ^21^, ^46^

These analyses had several strengths. First, results may be more generalizable than other studies because BCS were participants in a longitudinal study of mid‐aged women and not recruited for a sexual functioning study. Age at diagnosis ranged from 45.2 to 69.5, thus covering a wider aged range than studies focusing only on younger women. Although 20% of BCS and only 10% of controls were lost to follow‐up, which could have led to a healthy sample bias, this loss to follow‐up among BCS was much lower than that of prior longitudinal studies in which follow‐up rates ranged from 31% to 77.5% (most in the range of 31% to 46%).^1^, ^8^, ^27^, ^34^ The present study is thus likely to be less biased and have greater generalizability than prior studies. Second, controls came from the same cohort, reflecting the population from which BCS emanated. Third, SWAN assessed sexual functioning multiple times and for a longer period than previous studies, providing information about function prior to diagnosis and over a longer time since diagnosis. Finally, we investigated multiple domains of sexual functioning and a range of variables often associated with sexual functioning.

Several limitations of the study should be considered. First, the sexual functioning questionnaire was not one of the more newly developed, validated questionnaires, such as the SPEQ^43^ or FSFI.^41^ However, the score was developed based on items that mapped to the FSFI and compared favorably to the SPEQ.^42^ In addition, our sexual functioning questions inquired about the prior 6 months; thus, we did not examine sexual functioning immediately following diagnosis or treatment. Further, questions on chemotherapy and radiation treatment assessed ever/never received but not timing or duration, and we did not have information on type of surgery. Finally, the sample size of BCS provided limited statistical power to detect associations with sexual functioning, particularly for subgroups based on breast cancer treatments, such as SERMs and endocrine treatments, and/or change in MT stage, and to detect hypothesized effect modification by BCS‐control status.

In conclusion, in the first few years after diagnosis, among sexually active women, significantly more BCS reported vaginal dryness and pain with intercourse than did their counterparts without cancer. Being sexually active, frequency of sexual intercourse, and frequency of desire declined following diagnosis, but not at greater rates than those of women without cancer. Recent ASCO Guidelines provide recommendations for treating symptoms of vaginal and/or vulvar atrophy, such as dryness (which may reduce pain with intercourse), beginning with lubricants and vaginal moisturizers as a first option.^47^ Additional recommendations (e.g., low‐dose vaginal estrogen, pelvic floor physiotherapy, and topical analgesia) may be considered for patients who do not respond to first line options, or who have multifactorial reasons for pain. Psychosocial and/or psychosexual counseling should also be considered. Future research should examine longitudinally in a sufficiently large sample the specific impact of various cancer treatments (chemotherapy, radiation, endocrine therapies) on sexual functioning over time, as well as the distinct trajectories of change among a wide age range of survivors.

## AUTHOR CONTRIBUTIONS

**Nancy E. Avis:** Conceptualization (lead); formal analysis (supporting); funding acquisition (lead); investigation (lead); methodology (lead); project administration (lead); writing – original draft (lead); writing – review and editing (lead). **Sybil Crawford:** Conceptualization (supporting); data curation (lead); formal analysis (lead); funding acquisition (supporting); investigation (supporting); methodology (supporting); writing – original draft (supporting); writing – review and editing (supporting). **Ellen Gold:** Conceptualization (supporting); formal analysis (supporting); funding acquisition (supporting); investigation (supporting); methodology (supporting); writing – original draft (supporting); writing – review and editing (supporting). **Gail Greendale:** Conceptualization (supporting); formal analysis (supporting); funding acquisition (supporting); investigation (supporting); methodology (supporting); writing – original draft (supporting); writing – review and editing (supporting).

## FUNDING INFORMATION

The Study of Women's Health Across the Nation (SWAN) has grant support from the National Institutes of Health (NIH), DHHS, through the National Institute on Aging (NIA), the National Institute of Nursing Research (NINR) and the NIH Office of Research on Women's Health (ORWH) (Grants U01NR004061; U01AG012505, U01AG012535, U01AG012531, U01AG012539, U01AG012546, U01AG012553, U01AG012554, U01AG012495, and U19AG063720). Pink SWAN is supported by the National Cancer Institute R01CA199137. The content of this manuscript is solely the responsibility of the authors and does not necessarily represent the official views of the NIA, NINR, ORWH or the NIH.

## CONFLICT OF INTEREST

The authors do not have any conflicts of interest.

## Supporting information


Table S1

## Data Availability

The data that support the findings of this study are available from the corresponding author upon reasonable request.
