# Supplementary material for: Sexual functioning among breast cancer survivors and non‐cancer controls over 5 years post diagnosis: Pink SWAN
Source: Cancer Med. 2022 Nov 28;12(6):7356–68. doi: 10.1002/cam4.5433 (PMC10067058; doi:10.1002/cam4.5433)
Supplement: Supplementary file 1 — Table S1 [file CAM4-12-7356-s001.docx]

**Supplemental Table: Odds ratios (95% CI) for participant characteristics versus sexual functioning outcomes, adjusted for years since diagnosis/pseudo-diagnosis, combining cases and controls**

|  | | **Odds Ratio (95% CI)** | | | | | | | |
| --- | --- | --- | --- | --- | --- | --- | --- | --- | --- |
| **Characteristic** | | **Sexually active with partner in past 6 months; all visits** | | **Intercourse at least weekly in past 6 months; visits with concurrent partnered activity** | | **Desire at least weekly; all visits** | | **Any vaginal dryness in past 2 weeks; all visits** | |
| Concurrent menopause transition stage: | |  | |  | |  | |  | |
| Pre-/early  peri-menopause | | Reference | | Reference | | Reference | | Reference | |
| Late peri-  menopause | | **0.76 (0.62, 0.94)** | | 0.77 (0.57, 1.03) | | **0.62 (0.48, 0.82** | | 1.28 (0.98, 1.67) | |
| Post-menopause | | **0.53 (0.44, 0.63)** | | **0.60 (0.47, 0.76)** | | **0.42 (0.35, 0.51)** | | **1.54 (1.26, 1.89)** | |
| BSO / hysterectomy | | **0.64 (0.46, 0.90)** | | **0.46 (0.30, 0.73)** | | **0.62 (0.44, 0.88)** | | **1.58 (1.10, 2.27)** | |
| Menopausal HT | | 1.04 (0.78, 1.37) | | 0.73 (0.51, 1.05) | | 0.74 (0.53, 1.02) | | **1.45 (1.03, 2.04)** | |
| BC SERM or  endocrine  preparation | | **0.35 (0.15, 0.80)** | | **0.29 (0.12, 0.73)** | | **0.33 (0.13, 0.81)** | | 0.85 (0.35, 2.04) | |
| p-value | | **<.0001** | | **.0001** | | **<.0001** | | **.0004** | |
| Concurrent CES-D depressive symptoms | | **0.83 (0.71, 0.97)** | | 0.85 (0.66, 1.10) | | **0.77 (0.64, 0.94)** | | 1.09 (0.93, 1.27) | |
| p-value | | **.0199** | | .2104 | | **.0098** | | .2967 | |
| Concurrent any vaginal dryness | | **1.49 (1.31, 1.68)** | | 1.07 (0.90, 1.27) | | 1.08 (0.93, 1.24) | | -- | |
| p-value | | **<.0001** | | .4729 | | .3284 | |  | |
| Concurrent pain with intercourse at least sometimes | | -- | | **0.58 (0.47, 0.71)** | | **--** | | **--** | |
| **Supplemental Table Continued: Odds ratios (95% CI) for participant characteristics versus sexual functioning outcomes, adjusted for years since diagnosis/pseudo-diagnosis, combining cases and controls** | | | | | | | | | |
|  | | **Odds Ratio (95% CI)** | | | | | | | |
| **Characteristic** | | **Sexually active with partner in past 6 months; all visits** | | **Intercourse at least weekly in past 6 months; visits with concurrent partnered activity** | | **Desire at least weekly; all visits** | | **Any vaginal dryness in past 2 weeks; all visits** | |
| p-value | |  | | **<.0001** | |  | |  | |
| Concurrently very emotionally satisfied | | -- | | **2.14 (1.78, 2.58)** | | **--** | | **--** | |
| p-value | |  | | **<.0001** | |  | |  | |

| **Supplemental Table Continued: Odds ratios (95% CI) for participant characteristics versus sexual functioning outcomes, adjusted for years since diagnosis/pseudo-diagnosis, combining cases and controls (continued)** | | | |
| --- | --- | --- | --- |
|  | **Odds Ratio (95% CI)** | | |
| **Characteristic** | **Desire at least weekly; visits with concurrent partnered activity** | **Any vaginal dryness in past 2 weeks; visits with concurrent partnered activity** | **Pain with intercourse at least sometimes in past 6 months; visits with concurrent partnered activity** |
| Concurrent menopause transition stage: |  |  |  |
| Pre-/early  Peri-menopause | Reference | Reference | Reference |
| Late peri-menopause | 0.72 (0.52, 1.00) | 1.34 (0.90, 1.98) | **1.58 (1.05, 2.38)** |
| Post-menopause | **0.60 (0.47, 0.76)** | **2.25 (1.74, 2.91)** | **2.25 (1.69, 3.00)** |
| BSO / hysterectomy | 0.79 (0.51, 1.22) | **1.68 (1.06, 2.66)** | 1.63 (0.97, 2.75) |
| Menopausal HT | 0.76 (0.52, 1.10) | 1.43 (0.98, 2.07) | 1.16 (0.71, 1.90) |
| BC SERM or endocrine  preparation | **0.49 (0.18, 1.32)** | **2.21 (1.02, 4.78)** | **3.08 (1.29, 7.38)** |
| p-value | **.0016** | **<.0001** | **<.0001** |
| Concurrent CES-D depressive symptoms | **0.75 (0.58, 0.96)** | 1.16 (0.91, 1.48) | 1.29 (1.00, 1.66) |
| p-value | **.0243** | .2407 | .0513 |
| Concurrent any vaginal dryness | 0.86 (0.72, 1.02) |  | **3.22 (2.63, 3.93)** |
| p-value | .0897 |  | **<.0001** |
| Concurrent pain with intercourse at least sometimes | **0.52 (0.42, 0.64)** | **3.62 (2.94, 4.46)** | -- |
| p-value | **<.0001** | **<.0001** |  |
| Concurrently very emotionally satisfied | **2.06 (1.71, 2.48)** | **0.79 (0.67, 0.95)** | **0.69 (0.57, 0.84)** |
| p-value | **<.0001** | **.0122** | **.0002** |
